# Squalene epoxidase/SQLE is a candidate target for treatment of colorectal cancers with *p53* mutation and elevated c-*MYC* expression

**DOI:** 10.7150/ijbs.85724

**Published:** 2023-08-06

**Authors:** Yuyun Du, Matjaz Rokavec, Heiko Hermeking

**Affiliations:** 1 Experimental and Molecular Pathology, Institute of Pathology, Medical Faculty, Ludwig-Maximilians-Universität München, D-80337 Munich, Germany.; 2 German Cancer Consortium (DKTK), Partner site Munich, D-80336 Munich, Germany.; 3 German Cancer Research Center (DKFZ), D-69120 Heidelberg, Germany.

**Keywords:** SQLE/Squalene epoxidase, cholesterol synthesis, p53, miR-205, c-MYC, AP4, TFAP4, colorectal cancer, Terbinafine, therapy

## Abstract

Elevated expression of c-MYC and inactivation of *p53* represent two of the most common alterations in colorectal cancer (CRC). However, c-MYC and defective p53 are difficult to target therapeutically. Therefore, effectors downstream of both c-MYC and p53 may represent attractive, alternative targets for cancer treatment. In a bioinformatics screen we identified Squalene epoxidase/SQLE as a candidate therapeutic target that appeared to be especially relevant for cell survival in CRCs, which display elevated c-MYC expression and loss of p53 function. SQLE is a rate-limiting enzyme in the cholesterol synthesis. Here, we show that p53 supresses SQLE expression, cholesterol levels, and cell viability via the induction of *miR-205*, which directly targets *SQLE*. Furthermore, c-MYC induced *SQLE* expression directly and via its target gene *AP4*. The transcription factor AP4/TFAP4 directly induced *SQLE* expression and cholesterol levels, whereas inactivation of *AP4* resulted in decreased *SQLE* expression and caused resistance to Terbinafine, an inhibitor of SQLE. Inhibition of SQLE decreased viability of CRC cells. This effect was enhanced in CRCs cells with *p53* inactivation and/or enhanced c-MYC/AP4 expression. Altogether, our results demonstrate that SQLE represents a vulnerability for CRCs with *p53* inactivation and elevated c-MYC activity.

## Introduction

Colorectal cancer (CRC) is the third most commonly diagnosed malignancy and the second leading cause of cancer mortality, responsible for about 900.000 deaths world-wide annually 1. The 5-year survival rate of CRC patients is ~64% but drops to ~12% for metastatic CRC 2. Treatment consists of surgery, combined with radio-, chemo- and targeted therapy for advanced CRCs. Targeted therapy targets factors specifically altered in cancer cells or the tumor microenvironment to selectively impede tumor growth and spread while limiting damage to healthy cells 3. In contrast to other cancer types, such as breast cancer, where therapies targeting the estrogen receptor with Tamoxifen or the HER2-receptor with Herceptin are used in standard care, targeted therapy so far only resulted in marginal improvement of CRC patient survival. A reason for this may be that the appropriate targets for CRC have not been identified yet. Today, therapies targeting EGFR, BRAF, KRAS, HER2, NTRK and VEGF, as well as the immune-checkpoint inhibitors have been approved for treatment of a subset of metastatic CRC 4, 5. Therefore, the identification of novel, innovative targets for CRC treatment is of high relevance. Two of the most common alterations in CRC are the elevated/deregulated expression of c-*MYC* by mutations in the Wnt/APC/β-catenin pathway and inactivating mutations in *p53* 6. Therefore, the combination of both events represents a vulnerability specific for the majority of CRC cancers.

The oncogenic transcription factor c-MYC is a central driver in many human cancers, including CRC 7. The c-*MYC* gene represents a nodal point of many signaling pathways and is therefore ubiquitously up-regulated in cancer cells without having to be itself altered or mutated 6. In cancer cells, c-MYC drives the transition to proliferative and invasive adenocarcinomas that display a highly inflammatory, angiogenic and immune-suppressed stroma 8, 9. High c-MYC protein levels not only drive tumor initiation and progression, but are also essential for tumor maintenance, making c-MYC a highly attractive target for anti-cancer therapy 10. Permanent activation of Wnt signaling is one of the key events in CRC and is in most cases driven by the loss of *APC* 11. The *c-MYC* promoter is a direct target of the Wnt/β-catenin/Tcf4 signaling in CRC cells and is therefore consistently activated after mutation of *APC* or *β-catenin* 12. Deletion of *c-MYC* prevented the phenotypes of perturbed differentiation, migration, proliferation and apoptosis, which occur upon inactivation of *APC*, indicating that c-MYC represents a critical mediator of the early stages of neoplasia following *APC* loss 13. This suggests that inhibition of c-MYC may be an efficient therapeutic strategy for treating or preventing CRC. However, c-MYC does not represent an easily druggable protein, since it lacks enzymatic activity or any deep pocket, which could be targeted by small molecule inhibitors 14. Therefore, the inhibition of c-MYC regulators, co-factors, or factors down-stream of c-MYC that are required for the oncogenic functions of c-MYC is an attractive, alternative approach for the treatment of c-MYC-driven tumors.

The p53 protein mainly functions as a transcription factor that activates target genes, which mediate tumor suppression by inducing cell cycle arrest, cell death and senescence, as well as by suppressing glycolysis, EMT and numerous other processes relevant for tumor formation and progression 15. P53 is activated by diverse forms of cellular stress, which have in common that they lead to DNA damage: e.g. telomere attrition and oncogene-induced replication stress. The *p53* gene is the most frequently mutated gene in human cancer. In CRC, the mutation frequency of *p53* is about 60% 6 and almost 80% in metastatic CRCs 16. Since *p53* is a tumor suppressor, that is commonly inactivated/lost in tumors, it is hard to target therapeutically. An attractive, alternative approach for the treatment of tumors that harbor *p53* mutations is to target factors or processes that are induced/activated/de-repressed upon *p53* inactivation. Such factors/processes might represent vulnerabilities specific for cancer cells with *p53* inactivation.

In a bioinformatics screen we identified squalene epoxidase (SQLE) as a potential therapeutic target for CRCs with elevated c-MYC activity and inactivation of *p53*. SQLE is a rate-limiting enzyme in the synthesis of cholesterol 17. Tumor cell proliferation is highly dependent on sterol biosynthesis, and oncogenic processes altering this pathway likely represent a mechanism of metabolic adaptation, supporting rapid proliferation and survival of cancer cells 18. Here we show that c-MYC induces *SQLE* expression not only directly but to a large extent via the AP4 transcription factor. Furthermore, we identified miR-205 as a mediator of the repression of *SQLE* by p53. Finally, we show that pharmacological inhibition of SQLE suppresses CRC cell viability, which is more pronounced in *p53*-deficient CRC cells with high c-MYC expression, suggesting that SQLE represents a vulnerability of CRCs with *p53* inactivation and elevated c-MYC activity.

## Materials and Methods

### Cell culture, treatments and transfections

Cells were maintained in McCoy' 5A medium (Invitrogen, Carlsbad, CA, USA) supplemented with 10% fetal bovine serum (FBS) (Invitrogen) including 100 units/ml penicillin and 0.1 mg/ml streptomycin. All cells were cultivated in a 5% CO_2_ humidified incubator at 37°C. HCT116, RKO, and SW48 *p53*^-/-^ and *p53*^+/+^ cells were a gift from Bert Vogelstein (Johns Hopkins Medical School, Baltimore, Maryland, USA) 19. Human intestinal fibroblasts (CCD-18co) were obtained from ATCC (Wesel, Germany). The generation of *AP4*-deficient DLD1 and SW480 cells has been previously described 20. Doxycycline (DOX; Sigma, St Louis, MO) was dissolved in water (100 mg/mL stock solution) and applied at a final concentration of 100 ng/ml. To maintain cell pools harboring pRTR vectors 20, a final concentration of 8 μg/ml puromycin was used and added newly with fresh medium every two days. Nutlin-3a (Sigma) treatment was performed at a final concentration of 10 μM for 48 hours. The SQLE inhibitors Terbinafine hydrochloride (T8826) was purchased from Sigma-Aldrich and dissolved in DMSO at a stock concentration of 50 mM and stored at -20°C. Cholesterol (Sigma-Aldrich, #C3045) was dissolved in CHCl_3_ (stock concentration 10 mM) and the working concentration was 20 μg/ml. pRTR vectors were transfected with Lipofectamine® LTX & PLUS™ Reagent (Invitrogen). Generation of cell pools harboring pRTR vectors and containing over 80% of fluorescent marker protein-expressing cells was performed as described before 20 and included selection with Puromycin. MiRNA mimics, siRNAs and negative controls were transfected with Lipofectamine RNAiMAX Transfection Reagent (Invitrogen) according to manufacturer's instructions. FlexiTube siRNAs (consisting of a pool of 4 different siRNAs) targeting *SQLE* and *c-MYC*), control siRNAs, miR-205-5p mimics, antagomirs and controls were obtained from Qiagen (Hilden, Germany). Sequences are given in Supplemental Table 1.

### Western blot analysis

After lysis with RIPA lysis buffer (50 mM Tris/HCl pH 8.0, 250 mM NaCl, 1% NP40, 0.5% [w/v] sodium deoxycholate, 0.1% SDS), that contained protease inhibitors as well as PhosSTOP Phosphatase Inhibitor Cocktail Tablets (Roche), cell lysates were subjected to sonication for a 5-second period at the 20 kHz frequency and 20-minute centrifugation at 13,000 rpm and 4°C. Protein-containing supernatants were harvested for quantification with Pierce™ BCA Protein Assay Kit (Thermo Fisher Scientific). 30 μg protein aliquots were separated by 10% sodium dodecyl sulfate (SDS) polyacrylamide gel electrophoresis and transferred on Immobilon PVDF membranes (Millipore, Burlington, MA, USA) according to standard protocols (Bio-Rad Laboratories, Hercules, CA). For immunodetection membranes were incubated with antibodies listed in Supplemental Table 2. Signals from horse-radish-peroxidase (HRP)-coupled secondary antibodies were generated by enhanced chemiluminescence (Millipore) and recorded with a LI-COR Odyssey FC imaging system (Bad Homburg, Germany).

### Cell viability

1500 cells were seeded per well into 96-well plates in triplicates. Cell viability was determined using the Cell Counting Kit-8 (CCK-8, Dojindo) or CellTiter-Glo kits (Promega) according to manufacturer's instructions. Absorbance at 450 nm was determined using a Varioscan system (Thermo Fisher).

### Real‑time impedance measurements

Impedance measurements (Xcelligence RTCA DP, Roche) were performed to analyze cell proliferation. Cells (2 x 10^3^/well) were inoculated into 48-well microtiter plates (EPlate Cardio 96, Agilent, Santa Clara, CA, USA). Treatments were applied in triplicates and cell impedance was then recorded at 60-minute intervals for 200 hours using a X-celligence system (Roche) and analysed with RTCA software (Roche). Finally, a Neubauer chamber was used for counting cell at the endpoint to validate impedance measurements.

### RNA extraction and quantitative real‑time polymerase chain reaction (qPCR) assay

Total RNA was isolated using the High Pure RNA Isolation Kit (Roche) according to manufacturer's instructions. cDNA was prepared using 500 ng RNA and the Verso cDNA synthesis kit (Thermo Fisher Scientific, Waltham, MA). qPCR was conducted on a LightCycler 480 (Roche) system with Fast SYBR Green Master Mix (Applied Biosystems, Foster City, CA). *GAPDH* or *β-actin* was used as the internal reference for normalizing gene expression by the ΔΔCt approach 21. Results are represented as fold change after treatment or transfection relative to control. Each assay was carried out in triplicates. The sequences of oligonucleotides used as qPCR primers are listed in Supplemental Table 3.

### 3'-UTR reporter generation

An online miRNA prediction tool (Targetscan) was utilized to identify miR-205 binding sites in the 3'-UTR of *SQLE* mRNA. The full length 3'-UTR of human *SQLE* mRNA was amplified by PCR amplified from cDNA collected from SW48 cells. After inserting the PCR product in the pGL3-control-MCS vector it was validated by sequencing. For modification of the miR-205-5p seed-matching sequence (SMS) within the *SQLE* 3′-UTR we used the Mut Express® MultiS Fast Mutagenesis Kit V2 (Vazyme, China) according to manufacturer's instructions. The sequences of oligonucleotides used for cloning and mutagenesis are listed in Supplemental Table 4.

### Dual luciferase assay

H1299 cells (3 × 10^4^/well) were seed into 12-well plates. After 24 hours cells were transfected with specific pGL3 firefly luciferase reporter plasmids (100 ng), Renilla plasmid (20 ng, normalization control) and miR-205-5p mimic (25 nM, 339173, Qiagen) or negative control (NC) for 48 hours using HiPerFect Transfection Reagent (Qiagen). Afterwards, the Dual Luciferase Reporter assay kit (Promega) was employed to analyze luciferase activities using Orion II Microplate Luminometer (Berthold, Germany) and the Simplicity software package.

### Cholesterol content analysis

Cholesterol levels were detected in 10^6^ cells using a Cholesterol/Cholesterol Ester Quantification kit (ab65359, Abcam) according to manufacturer's instructions. The assay was carried out in triplicates.

### Chromatin immunoprecipitation assay

Chromatin immunoprecipitation (ChIP) was performed in SW480 cells using the iDeal ChIP-qPCR kit (Diagenode, Belgium) according to manufacturer's instruction. Antibodies and sequences of qChIP primers are provided in Supplemental Table 2 and 5, respectively.

### Bioinformatics analysis of public datasets

Cancer cell dependency datasets and characteristics for CRC cell lines were retrieved from the Cancer Dependency Map (DepMap; https://depmap.org/portal/). c-MYC- and p53-regulated genes were identified based on datasets from the NCBI Gene Expression Omnibus (GEO). The regulation of the expression of these genes was assessed using gene expression profiling datasets of cell lines/tissues with ectopic expression or knockdown (KD) of c-MYC or treatment with p53-inducing compounds. Expression and clinical data of the TCGA colon adenocarcinoma (COAD) and rectal adenocarcinoma (READ) cohorts was obtained from the MD Anderson standardized data browser (http://bioinformatics.mdanderson.org/TCGA/databrowser/). The RNA-Seq by Expectation-Maximization (RSEM) normalized expression values from the Illumina RNASeqV2 (genes) datasets were used. Expression and clinical data of other CRC patient datasets was downloaded from NCBI GEO (www.ncbi.nlm.nih.gov/geo). Differential expression between tumors and adjacent normal colon tissue was calculated by paired t-test. Differential expression between tumors of different stages was calculated using 1-way ANOVA with a post-test for linear trend from stage 1 to stage 4. The statistics for survival analysis was calculated by log-rank test. For binary classification of cases (high/low expression), the Survminer R-package (https://CRAN.R-project.org/package=survminer) was used to determine optimal cutoff values. The CMS and CRIS classification of public datasets was obtained from Guiney et al 22 and Isella et al 23.

### Statistical analysis

Correlations were determined using the Pearson correlation coefficient and two-tailed p-value provided by GraphPad prism 7.0. Correlations with p-values less than 0.05 were considered significant. The statistical differences between two groups were calculated using a Student's t test (two-tailed; unpaired). One-way analysis of variance (ANOVA) with the Tukey multiple comparison post-test was used to compare more than 2 groups. The results are represented as the mean ± SD. All statistical analyses were performed with the GraphPad Prism software 7.0. p-values of less than 0.05 were considered significant and indicated by asterisks (*, p< 0.05; **, p < 0.01; ***, p < 0.0001 or ****, p < 0.00001).

## Results

### Identification of therapeutic targets in p53 mutant CRCs with elevated c-MYC expression

**Figure 1:**
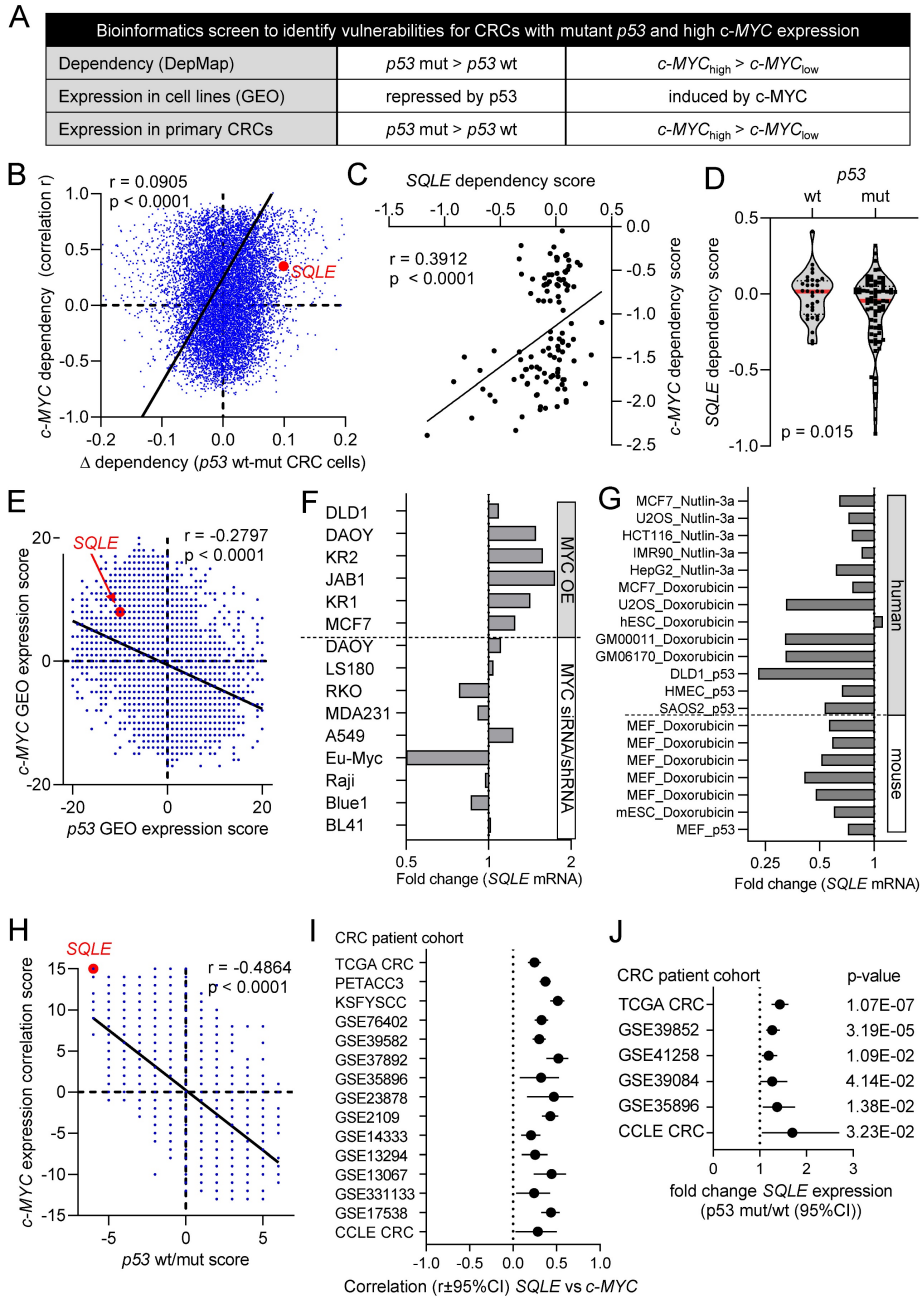
**Bioinformatics identification of therapeutic targets in CRCs with mutant *p53* and high c-*MYC* expression. (A)** Schematic representation of the bioinformatics screen.** (B)** Correlation of *c-MYC*- and p53-related dependencies in CRC cell lines. Lower dependency scores correspond to higher dependency. *SQLE* is marked in red. See results for detailed explanation. **(C)** Correlation between *SQLE* and *c-MYC* dependencies in CRC cell lines. **(D)** *SQLE* dependency in *p53* wt and mutant CRC cell lines. **(E)** Correlation of c-*MYC* and *p53* GEO expression scores in cell lines. *SQLE* is marked in red. See results for detailed explanation. **(F)** Fold changes in *SQLE* expression in GEO datasets representing studies with c-*MYC* ectopic expression or knockdown in the indicated cell lines. **(G)** Fold changes in *SQLE* expression in GEO datasets representing studies with p53 activation in indicated cell lines. **(H)** Correlation of c-*MYC* expression and *p53* wt/mut scores in primary CRCs. *SQLE* is marked in red. See text for detailed explanation. **(I)** Correlation between *SQLE* and *MYC* mRNA expression in CRCs from the indicated cohorts. **(J)** *SQLE* expression in *p53* mutant vs *p53* wt primary tumors from indicated CRC patient cohorts.

To identify vulnerabilities of CRCs with mutant *p53* and elevated c-MYC expression we performed a comprehensive bioinformatics analysis of multiple, large patient- and cell-line derived datasets (Figure 1A). First, we analyzed CRC cell line data from the Cancer Dependency Map (DepMap) to identify genes, that show co-dependencies with c-MYC and display stronger dependencies in *p53* mutant CRC cell lines when compared to *p53* wt cells. For each gene, we calculated the correlation coefficient between the dependency on the gene and the dependency on c-MYC across CRC cell lines. The goal was to identify positively correlated genes, which would indicate that cell lines with high dependency on c-MYC also show high dependency on the gene of interest. Furthermore, we calculated the difference in dependency for each gene in *p53* wt vs. *p53* mutant CRC cell lines. The integration of the c-MYC- and p53-specific differential dependencies revealed a significant positive correlation between the global c-MYC- and *p53*-mutant-related gene dependencies (Figure 1B). Therefore, our results suggest that genes that exert co-dependencies with c-MYC show higher dependencies in *p53* mutated CRC cell lines, and vice versa. This screen identified 116 genes that show a significant co-dependency with c-MYC and a significant higher dependency in *p53* mutated CRC cell lines (Rokavec et al., manuscript in preparation). *SQLE* was among the genes whose dependency in CRC cell lines showed the most significant positive correlation with the dependency on c-MYC, indicating that cell lines that are highly dependent on c-MYC also show high dependency on SQLE (Figure 1C). Furthermore, *p53* mutant CRC cell lines showed a significantly higher dependency on SQLE than *p53* wt cells (Figure 1D).

Next, we performed a meta-analysis of twenty public Gene Expression Omnibus (GEO) datasets representing studies of ectopic expression or knockdown/knockout of *c-MYC* in cell lines or mice to identify genes that are regulated by c-MYC. For each study a score of +1 was assigned to genes that were significantly induced after ectopic expression of *c-MYC* or repressed after *c-MYC* knockdown, whereas a score of -1 was assigned to genes that were repressed after ectopic *c-MYC* or induced after *c-MYC* knockdown. Scores for each gene in every study were summed up to obtain a c-MYC GEO expression score.

This score indicates the number of studies in which a gene was induced or repressed by c-MYC. Thus, the size of the score indicates the consistency of the regulation by c-MYC. To identify genes that are regulated by p53, we utilized the data from a similar meta-analysis of twenty GEO datasets representing studies with p53 activation in cell lines 24 and thereby obtained a p53 GEO expression score. Interestingly, the integration of c-MYC and p53 GEO expression scores revealed a significant inverse correlation between the global gene regulation exerted by c-MYC and p53, indicating that many genes that are induced by c-MYC are repressed by p53 and vice versa (Figure 1E). *SQLE* mRNA was induced after ectopic expression of c-MYC and down-regulated after repression of c-MYC in the majority of GEO studies (Figure 1F). Furthermore, *SQLE* mRNA was suppressed after activation of p53 in the majority of GEO studies with human and mouse cell lines (Figure 1G).

Finally, we performed a similar meta-analysis in primary CRCs. For each gene we calculated the correlation coefficient between its expression and the expression of *c-MYC* in primary tumors from 15 CRC patient cohorts. For each cohort, genes that showed a significant positive or negative correlation with *c-MYC* were assigned with a score of +1 and -1, respectively. Scores for each gene in every cohort were summed up to obtain a c-MYC expression correlation score, which indicates the number of studies in which a gene was significantly positively or negatively correlated with the expression of *c-MYC*. To determine p53-regulated genes in CRCs, we identified genes that were differentially expressed between *p53* wt and mutant primary tumors from six CRC patient cohorts. Scores were assigned to genes indicating the number of cohorts in which the expression of a gene is higher in *p53* wt tumors (positive score) or higher in *p53* mutant tumors (negative score). Similar to GEO expression scores from cell lines, also c-MYC- and p53-scores from primary tumors showed a strong inverse correlation, suggesting that genes that positively correlate with the expression of *c-MYC* are higher expressed in *p53* mutant tumors and those lower in c-MYC low tumors are low in *p53* wt tumors (Figure 1H). The expression of *SQLE* mRNA showed a strong and consistent positive correlation with *c-MYC* mRNA expression in all 15 cohorts of primary CRCs and CRC cell lines (Figure 1I). Furthermore, expression of *SQLE* mRNA was consistently higher in tumors with mutant *p53* in all six cohorts of primary CRCs and CRC cell lines (Figure 1J).

### Association of SQLE expression with clinico-pathological parameters

**Figure 2:**
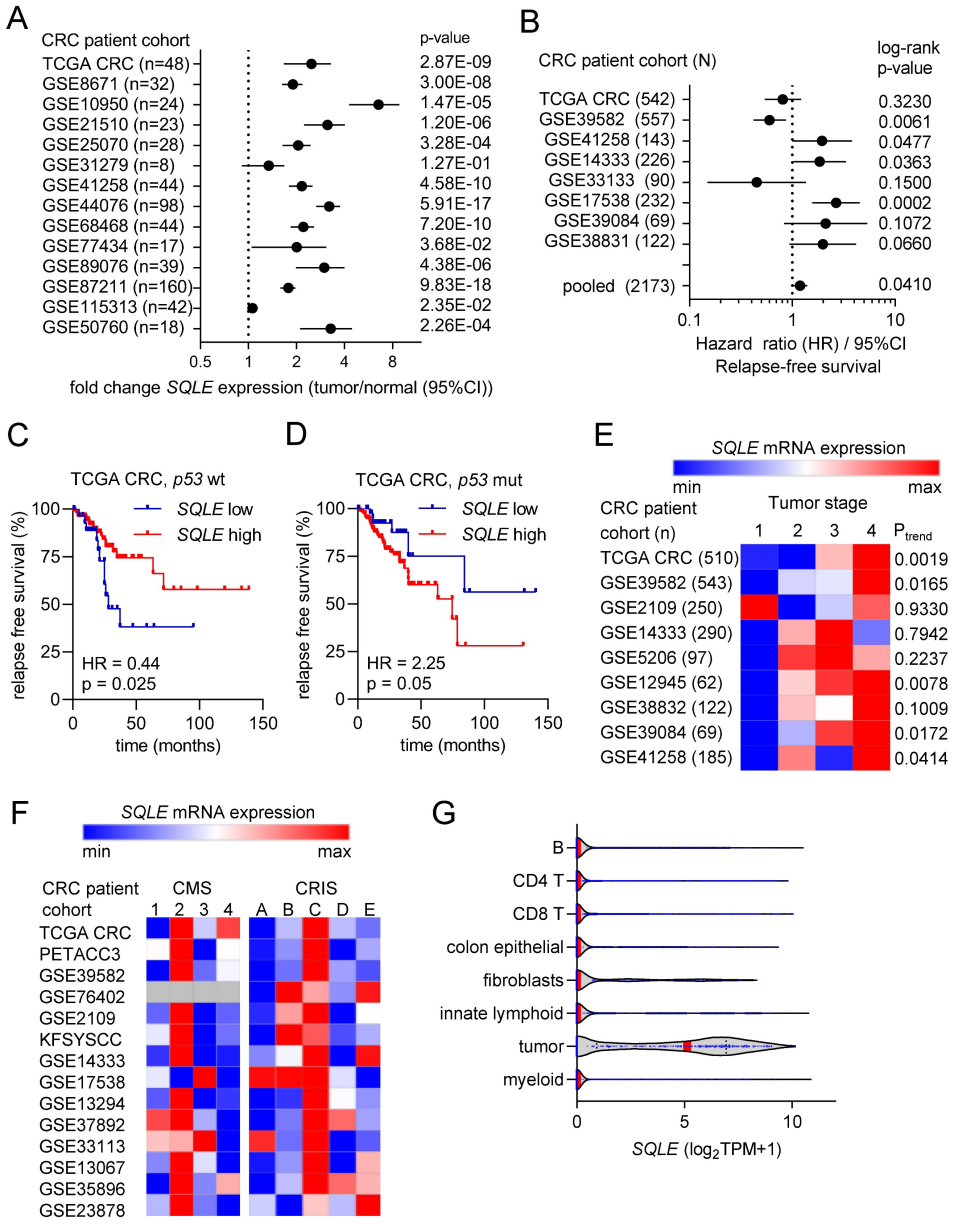
**Associations of *SQLE* expression with clinical and pathological characteristics in CRC. (A)** Forest plot showing fold changes in *SQLE* expression between colorectal tumors and matched adjacent normal colonic mucosa in patient cohorts. Dots represent fold changes and horizontal lines show 95% CI. Significance was determined using paired t-test. **(B)** Forest plot showing hazard ratios for relapse free survival by comparing patients with high versus low expression of *SQLE* in CRC patient cohorts. Dots represent hazard ratios and horizontal lines show 95% CI. P-values were calculated using the log-rank method. **(C and D)** Kaplan-Meier analysis of RFS according to the *SQLE* expression in TCGA *p53* wt (C) and *p53* mutant (D) CRC cohorts. **(E)** Associations of *SQLE* expression with tumor stage. Significance was determined using one-way ANOVA with a post-test for linear trend from stage 1 to stage 4. **(F)** *SQLE* expression in different cancer molecular subtypes (CMS) 19 and colorectal cancer intrinsic subtypes (CRIS). **(G)** Expression of *SQLE* mRNA in the indicated cell types present within colon tumors (data from single cell RNA-Seq (GSE81861)).

Next, we analyzed the association of *SQLE* mRNA expression with clinico-pathological parameters in publicly available CRC patient cohorts. *SQLE* mRNA expression was strongly and consistently elevated in colorectal tumors compared to adjacent normal tissue in 13 out of 14 patient cohorts (Figure 2A). Furthermore, high expression of *SQLE* was significantly associated with poor survival in an analysis of 8 pooled CRC patient cohorts (Figure 2B). Interestingly, stratification of patients according to the *p53* mutational status of CRCs revealed that in CRCs with wt *p53* high *SQLE* expression was associated with good survival (Figure 2C), whereas in CRCs with mutant *p53* high *SQLE* expression was associated with poor patient survival (Figure 2D). Moreover, *SQLE* expression progressively increased from stage 1 to stage 4 primary tumors in 5 out of 9 CRC patient cohorts (Figure 2E). Next, we analyzed *SQLE* expression in different cancer molecular subtypes (CMS) 22 and colorectal cancer intrinsic subtypes (CRIS) 23. *SQLE* mRNA levels were highest in CMS2 and in CRIS C/D, which are molecular sub-types of CRC characterized by high Wnt/c-MYC activity and intestinal stem cell gene expression (Figure 2F). Finally, analysis of single cell RNA-Seq data from primary CRCs 25 showed that *SQLE* mRNA is predominantly expressed in tumor cells when compared to normal colon epithelial and stromal cells (Figure 2G), suggesting that therapeutic targeting of *SQLE* may have weak effects in non-tumor cells.

### Suppression of SQLE decreases viability of CRC cells

**Figure 3:**
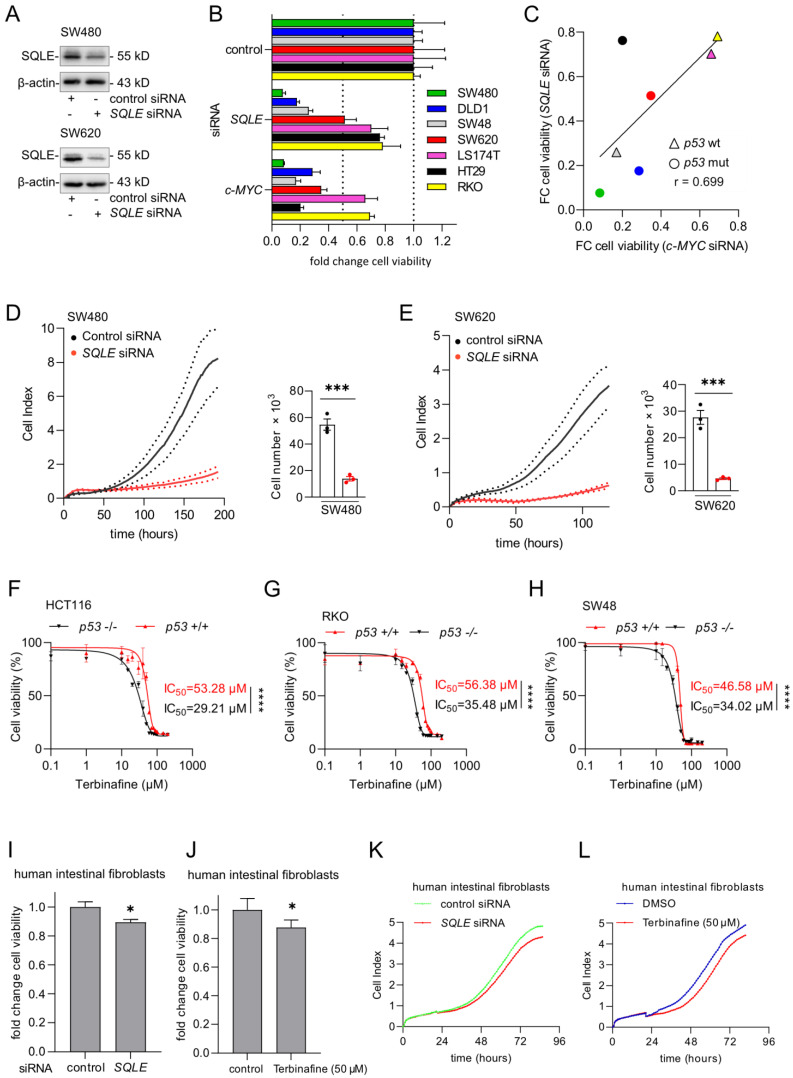
**Suppression of SQLE results in decreased viability of CRC cells. (A)** Western blot analysis of indicated proteins in SW480 and SW620 cells after transfection of indicated siRNAs for 48 hours. **(B)** 48 hours after transfection with siRNAs cells were transferred into new 96-well plates (5000 cells/well) and transfected again with siRNAs. After 5 days cell viability was analyzed with CellTiter GLO. **(C)** Correlation between the fold changes in cell viability between cells transfected with *SQLE* siRNA and *c-MYC* siRNA. Color coding as in panel B. **(D)** and **(E)** Left panels: Cell proliferation was determined by impedance measurement after transfection with siRNAs. Right panels: Cell numbers were determined using a Neubauer chamber at the last time point.** (F-H)** Cell viability analysis after inhibition of SQLE with the increasing concentration of Terbinafine for 96 hours in three pairs of syngeneic *p53*-proficient and *p53*-deficient CRC cell lines. **(I and J)** Cell viability analyzes of human intestinal fibroblasts transfected with indicated siRNAs (I) or treatment with Terbinafine for 96 hours (J)**. (K and L).** Cell proliferation of human intestinal fibroblasts determined by impedance measurement after transfection with siRNAs or treatment with Terbinafine (L). In panels A, F- J, the mean ± SD (n = 3) is provided. *: p < 0.05, **: p < 0.01, ***: p < 0.001.

To determine whether a therapeutic inhibition of *SQLE* may reduce CRC cell viability and proliferation, we suppressed *SQLE* using a pool of *SQLE*-specific siRNAs (Figure 3A). Knockdown of *SQLE* decreased cell viability in seven CRC cell lines (Figure 3B). The silencing of *SQLE* resulted in a similar suppression of cell viability as silencing of *c-MYC* in all cell lines (Figures 3B and 3C). This confirmed our previous, bioinformatics-based observation (Figure 1C), which indicated that cell lines, which are highly dependent on c-MYC also show a high dependency on SQLE.

The suppression of *SQLE* in SW480 and SW620 CRC cells resulted in a strong decrease of proliferation as determined by measuring cellular impedance and cell number evaluation at the end-point (Figures 3D and 3E). Next, we used the SQLE-inhibitor Terbinafine to treat three pairs of syngeneic *p53*-proficient and *p53*-deficient CRC cell lines. Analysis of cell viability showed that *p53*-deficient cells were more sensitive to the Terbinafine than *p53*-proficient cells (Figure 3F-H), which is consistent with the bioinformatics analysis of DepMap data showing that *p53*-mutant CRC cell lines display a higher dependency on SQLE than *p53* wt cells (Figure 1D). In the absence of Terbinafine, *p53*-deficient HCT116, RKO, and SW48 cells showed an increased cell viability when compared to their *p53*-proficient counterparts (Supplemental Figure 1A-C). Finally, knockdown of *SQLE* by siRNA or inhibition of SQLE by Terbinafine resulted in an only minor suppression of cell viability and impedance/proliferation of normal human intestinal fibroblasts (Figure 3I-L and Supplementary Figures 2A, B), indicating that the inhibition of SQLE may have minor effects on non-tumor cells and therefore presumably exhibit less side-effects on the organismal level.

### p53 represses SQLE expression and cholesterol synthesis

**Figure 4:**
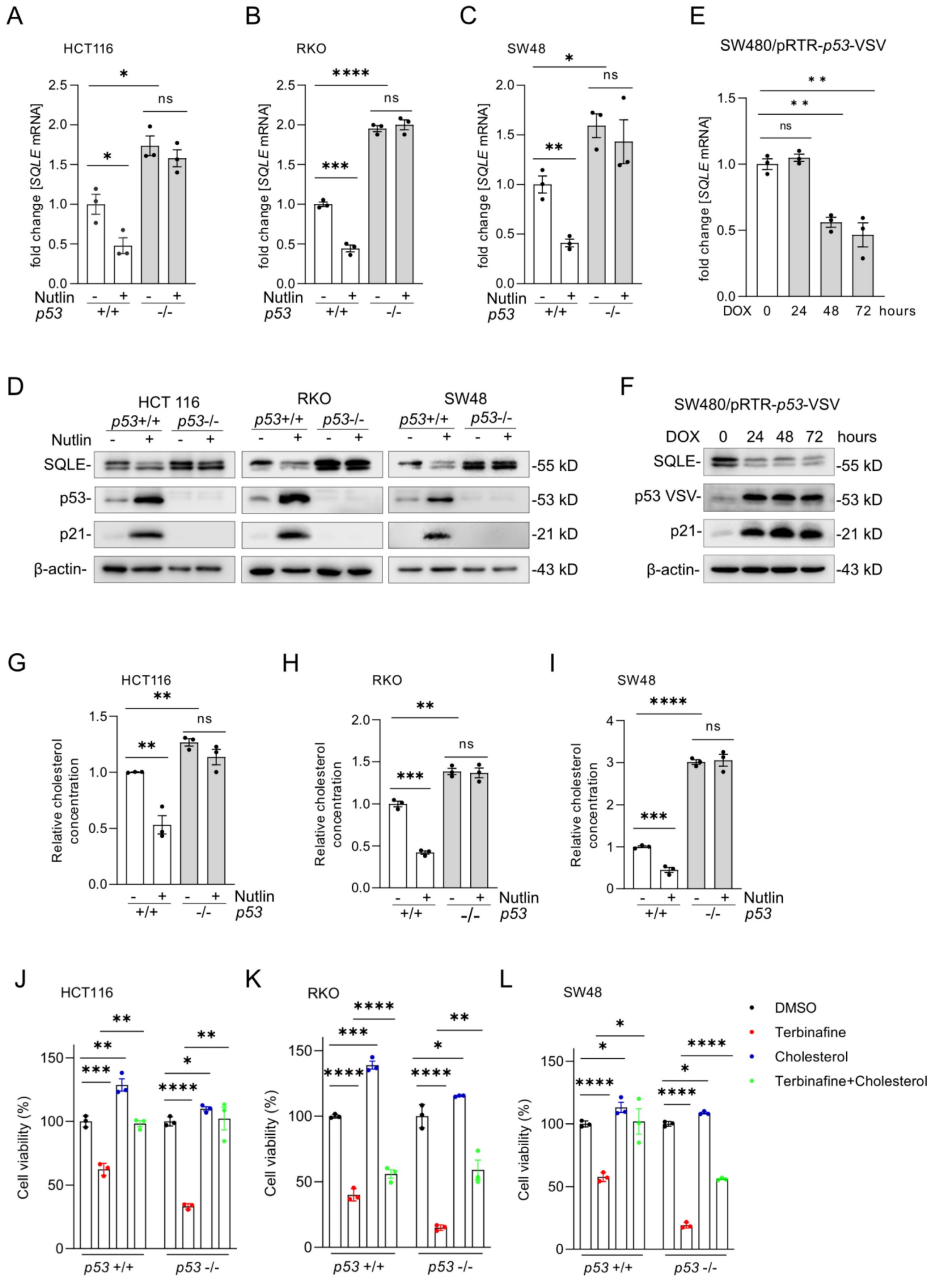
**p53 activation represses *SQLE* expression and cholesterol synthesis. (A-C)** qPCR analysis of *SQLE* expression after activation of p53 by Nutlin-3a (10 µM) for 48 hours. **(D)** Western blot analysis of SQLE, p53 and p21 expression after Nutlin-3a treatment. **(E-F)** qPCR and Western blot analysis in SW480 cells after induction of ectopic wt *p53* by DOX for the indicated periods.** (G-I)** Cholesterol levels in the indicated cells after activation of p53 via Nutlin-3a. **(J-L)** Cell viability of indicated cells after treatment with Terbinafine and/or Cholesterol. In panels of A-C, E, G-L, the mean ± SD (n=3) is provided. *p < 0.05, **p < 0.01, and ***p < 0.001.

Our bioinformatics results from public datasets had suggested that *SQLE* is repressed by p53 (Figure 1G). For experimental validation, we treated the syngeneic pairs of *p53* wt and *p53* knockout HCT116, RKO and SW48 CRC cell lines with the p53-activator Nutlin-3a, a small-molecule compound antagonizing the inhibitory interaction of MDM2 with p53 26. After activation of p53 by Nutlin-3a, *SQLE* mRNA (Figures 4A-C) and protein expression (Figure 4D) was significantly repressed in *p53* wt cells, but not in *p53* knockout cells. Of note, *SQLE* expression was higher in untreated *p53* knockout cells when compared to *p53* wt cells. Furthermore, we analyzed the expression of *SQLE* in SW480 cells with DOX-inducible ectopic expression of wt p53, which we had previously established 27. After DOX treatment the mRNA and protein expression of SQLE was repressed (Figure 4E and F). Next, we examined relative cholesterol levels after p53 activation. After treatment with Nutlin-3a, cholesterol levels were significantly repressed in *p53* wt cells, but not in *p53* knockout cells (Figures 4G-I). Moreover, *p53* knockout cells showed increased cholesterol levels when compared with *p53* wt cells. Next, we treated *p53* wt and *p53* knockout HCT116, RKO, and SW48 cells with the SQLE inhibitor Terbinafine and/or cholesterol. Cholesterol substitution completely rescued cell viability in Terbinafine treated *p53*-proficient and *p53*-deficient HCT116 cells (Figure 4J). In RKO the rescue was partial irrespective of the *p53* status (Figure 4K) and in SW48 the rescue was complete in *p53*-proficient and partial in *p53*-deficient cells (Figure 4L). Altogether, our results show that expression of SQLE and cholesterol levels are suppressed by p53. Furthermore, the resulting decrease in cholesterol results in decreased viability of CRC cells.

### SQLE is a miR-205 target

**Figure 5:**
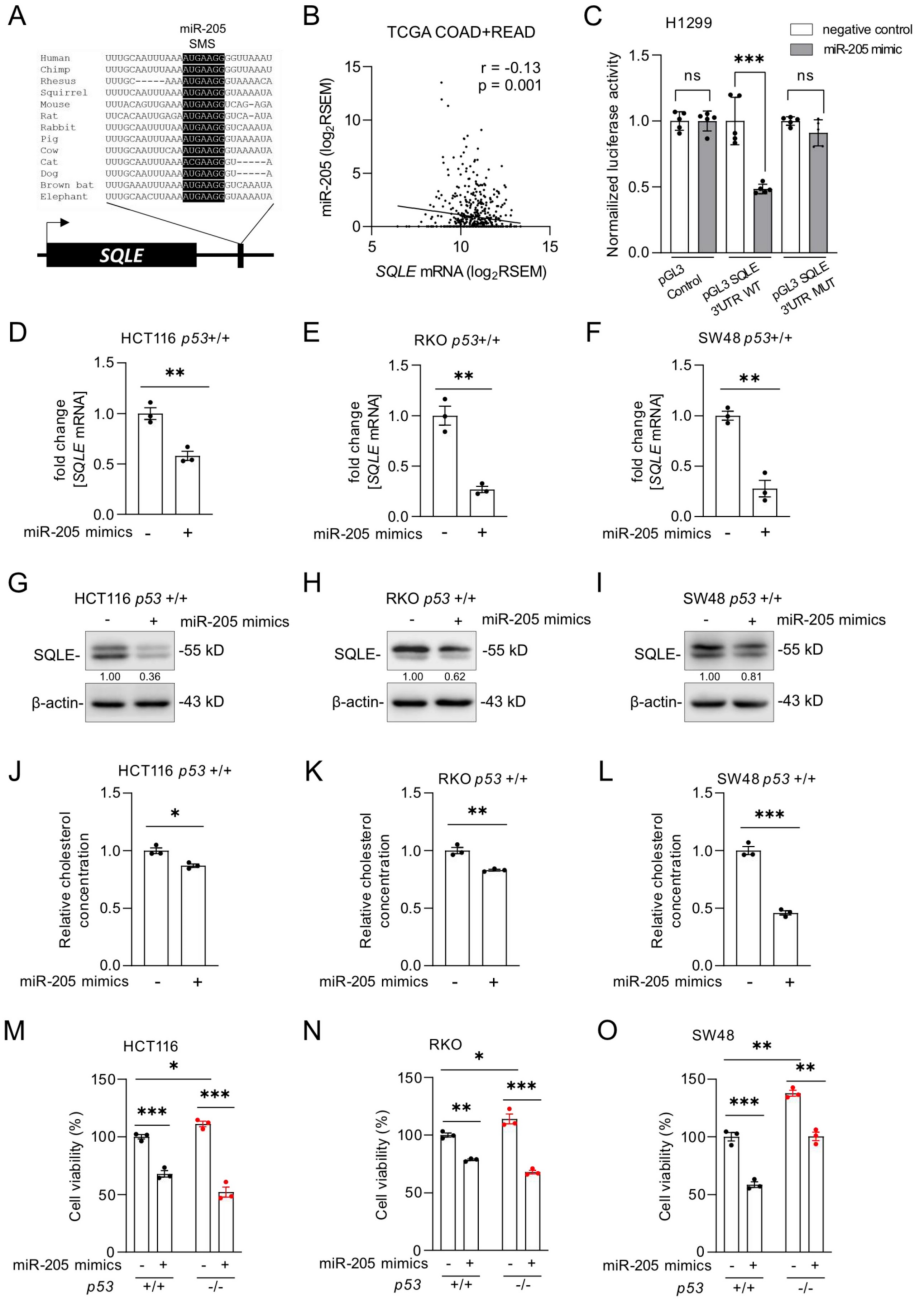
***SQLE* represents a target of miR-205. (A)** Schematic representation of the *SQLE* 3'-UTR indicating the *miR-205* seed-matching sequence (SMS; highlighted in black) and its phylogenetic conservation. **(B)** Correlation between *SQLE* and *miR-205* expression in TGCA colon adenocarcinomas (COAD) and rectum adenocarcinomas (READ). **(C)** Dual-luciferase assay was conducted 48 hours after transfection of H1299 cells with miR-205 mimics and human *SQLE* wt or mutant 3'-UTR reporters. Data resents the mean ± SD of five replicates. **(D-F)** qPCR analysis of *SQLE* expression after transfection of *miR-205* mimics four 48 hours. **(G-I)** Western blot analysis after transfection of miR-205 mimics for 48 hours. **(J-L)** Relative cholesterol concentration after transfection of miR-205 mimics for 72 hours. **(M-O)** Cell viability after transfection of *miR-205* mimics for 72 hours. In panel D-F and J-O the mean ± SD (n = 3) is provided. *: p < 0.05, **: p < 0.01, ***: p < 0.001, ****: p < 0.0001.

p53 does not repress its target genes directly, but indirectly via microRNAs (miRNA) or the DREAM complex 28. According to a meta-analysis, *SQLE* is not a target of the DREAM complex 29. MiRNAs induced by p53 have been previously shown to participate in the regulation of cell proliferation and apoptosis by suppressing the expression of important oncogenes 30. We identified a conserved sequence in the 3'-UTR of the *SQLE* mRNA that matches the seed region of miR-205, which is a *p53*-inducible miRNA 31 (Figure 5A). Therefore, p53 might suppress *SQLE* via the induction of miR-205. The expression of *SQLE* and miR-205 expression showed a significant negative correlation in the TGCA dataset of primary CRC tumors (r =-0.13, p=0.001; Figure 5B). Dual luciferase assays confirmed that ectopic miR-205 directly suppresses the *SQLE* 3'-UTR mediated enhancement of translation, which was abolished by mutations of the miR-205 binding site (Figure 5C). Since luciferase reporter assays may have limited physiological relevance, we also determined the effect of ectopic miR-205 on endogenous *SQLE*. After transfection of miR-205 mimics, which are synthetic RNAs that simulate naturally occurring mature miR-205, *SQLE* mRNA levels were significantly decreased in HCT116, RKO, and SW48 cells (Figure 5D-F). Also, SQLE protein was decreased (Figure 5G-I). Consistently, ectopic miR-205 also suppressed cholesterol levels (Figure 5J-L) and cell viability (Figure 5M-O) in HCT116, RKO, and SW48 cells.

### p53 represses SQLE expression and cholesterol levels via the induction of miR-205

To verify that miR-205 expression is induced in a p53-dependent manner in CRC cell lines, we treated three pairs of syngeneic *p53*-proficient and *p53*-deficient CRC cell lines with Nutlin-3a.

**Figure 6:**
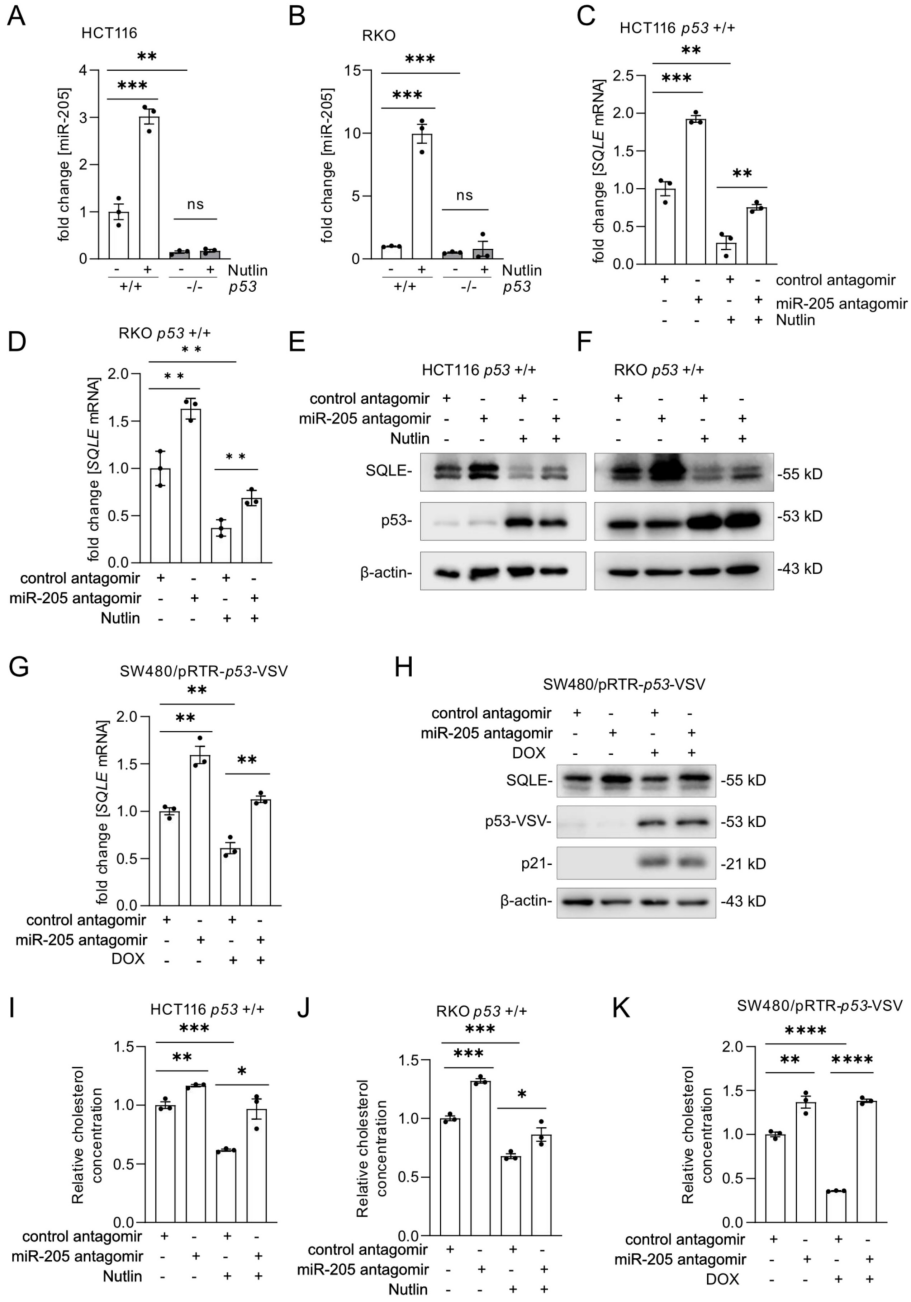
***SQLE* is indirectly repressed by p53 via *miR-205*. (A-B)** qPCR analysis of *SQLE* mRNA expression after the activation of p53 by Nutlin-3a. **(C-D)** qPCR analysis of *SQLE* expression transfection of *miR-205* antagomirs for 60 hours and Nutlin-3a treatment for 48 hours. **(E-F)** Western blot analysis of SQLE expression after transfection of *miR-205* antagomir for 60 hours and Nutlin-3a treatment for 48 hours. **(G-H)** qPCR (G) and Western blot (H) analysis of SQLE mRNA and protein expression after transfection of *miR-205* antagomir for 60 hours and activation of ectopic wt *p53* expression by treatment with DOX for 48 hours. **(I-J)** Relative cholesterol concentration in cells treated as in **(C). (K)** Relative cholesterol concentration in indicated cells treated as in (G)**.** In panel A-D, G and I-K the mean ± SD (n = 3) is provided. *: p < 0.05, **: p < 0.01, ***: p < 0.001, ****: p < 0.0001.

After activation of p53 by Nutlin-3a, miR-205 expression was significantly induced in *p53* wt cells, but not in *p53* knockout cells (Figure 6A-B and Supplemental Figure 3A). Next, miR-205 was inhibited with antagomirs. Expression of *SQLE* mRNA and protein was up-regulated after miR-205 inhibition (Figure 6C-F and Supplemental Figure 3B-C). Importantly, the suppression of *SQLE* mRNA and protein by p53 could be reverted by miR-205 antagomirs (Figure 6 C-F and Supplemental Figure 3B-C). Similarly, the inhibition of miR-205 with antagomirs reverted the down-regulation of *SQLE* mRNA and protein by ectopic expression of wt p53 in SW480/pRTR-*p53*-VSV cells (Figure 6G-H). In agreement with its effect on *SQLE* expression, inhibition of miR-205 completely abolished the decrease in cholesterol levels after p53 activation in HCT116 and RKO cells as well as after expression of ectopic p53 in SW480/pRTR-*p53*-VSV cells (Figure 6I-K). Altogether, our results show that the suppression of *SQLE* expression and cholesterol levels by p53 is mediated by the induction of miR-205.

### SQLE expression is induced by c-MYC

**Figure 7:**
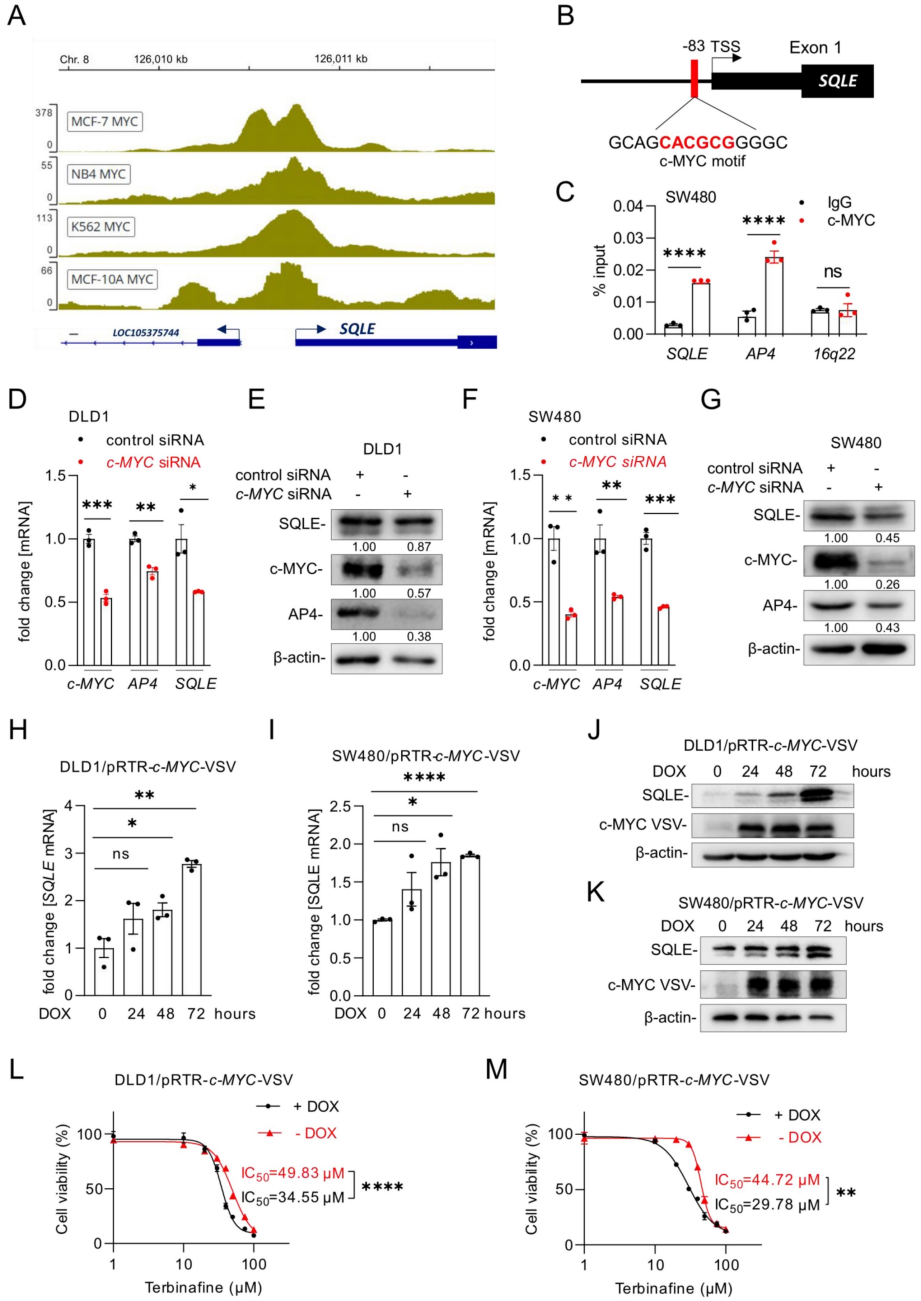
** Characterization of *SQLE* as a c-MYC target. (A)** c-MYC ChIP-Seq signal at the *SQLE* promoter in the indicated cell lines. Numbers on the y-axis indicate ChIP-Seq reads. **(B)** Schematic representation of the *SQLE* promoter region indicating the *c-MYC* binding site. **(C)** qChIP analysis of c-MYC occupancy at the human *SQLE* genomic region in SW480 cells. *AP4* and *16q22* served as positive and negative controls, respectively. **(D)** qPCR and **(E)** Western blot analysis after transfection of DLD1 cells with the indicated siRNAs. **(F)** qPCR and **(G)** Western blot analysis after transfection of SW480 cells with siRNAs. **(H-I)** *SQLE* mRNA levels in DLD1 (H) and SW480 (I) cells after c-*MYC* induction by DOX for the indicated periods. **(J-K)** SQLE protein levels in DLD1 (J) and SW480 (K) cells after c-*MYC* induction by DOX for the indicated periods. **(L-M)** Cell viability analysis after inhibition of SQLE with the increasing concentration of the SQLE-inhibitor Terbinafine for 96 hours in DLD1 (L) and SW480 (M) cells with or without DOX treatment. In panel C, D, F, H, I, L and M the mean ± SD (n = 3) is provided. *: p < 0.05, **: p < 0.01, ***: p < 0.001, ****: p < 0.0001. TSS: Transcription start site.

Our analysis of the Encode ChIP-Seq data from multiple cell lines showed that c-MYC binds to the promoter region of *SQLE* near the transcription start site (TSS) (Figure 7A). Furthermore, we identified a putative c-MYC binding site in the *SQLE* promoter region (Figure 7B). A qChIP assay confirmed enhanced c-MYC occupancy at this binding site within the *SQLE* promoter in SW480 cells (Figure 7C). As mentioned above, our analysis of public datasets suggested that the expression of *SQLE* is induced by c-MYC (Figure 1F). To validate this regulation, we suppressed c-MYC in DLD1, SW480, and SW620 cells with a pool of c-*MYC*-specific siRNAs. *SQLE* and *AP4*, a known c-MYC target 32, mRNA and protein levels decreased after silencing of *c-MYC* (Figure 7D-G, Supplemental Figure 4A-B). Conversely, ectopic expression of c-MYC induced *SQLE* mRNA and protein expression in DLD1 and SW480 cells (Figure 7H-K). Next, we used the SQLE-inhibitor Terbinafine and measured cell viability after ectopic c-MYC expression. While Terbinafine reduced the viability of DLD1 and SW480 cells, ectopic c-MYC expression further increased the sensitivity of these cells to Terbinafine (Figure 7L and M). Therefore, c-MYC up-regulates *SQLE* expression and increases the sensitivity towards SQLE inhibitors in CRC cells.

### Regulation of *SQLE* expression by AP4

**Figure 8:**
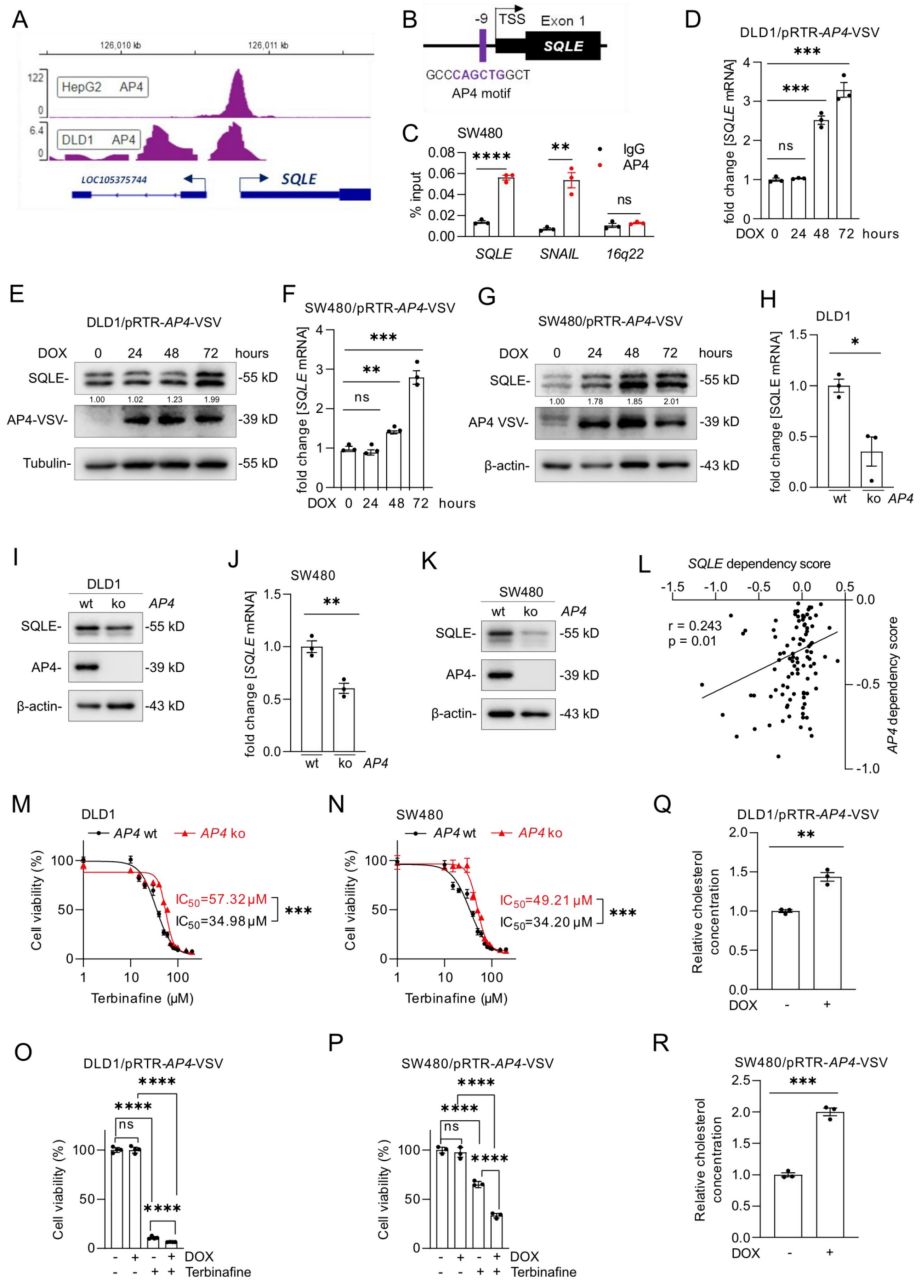
***SQLE* is directly induced by AP4. (A)** AP4 ChIP-seq signal at the *SQLE* promoter in the indicated cell lines. Numbers on the y-axis indicate ChIP-Seq reads. **(B)** Schematic representation of the *SQLE* promoter region indicating a predicted AP4 binding site. **(C)** qChIP analysis of AP4 occupancy at the human *SQLE* promoter region in SW480 cells. *SNAIL* and *16q22* served as positive and negative controls, respectively.** (D-G)** qPCR (D, F) and Western blot (E, G) analysis of SQLE expression after *AP4* induction by DOX for the indicated periods in (D,E) DLD1 and (F,G) SW480 cells. **(H-K)** qPCR (J) and Western blot (K) analysis in *AP4*-proficient and *AP4*-deficient (H,I) DLD-1 and (J,K) SW480 cells. (**L**) Correlation between *SQLE* and *AP4* dependencies in CRC cell lines (data from the cancer dependency map).** (M-N)** Cell viability analysis after inhibition of SQLE with the increasing concentration of Terbinafine for 96 hours in *AP4*-proficient and *AP4*-deficient cells DLD1 (M) and SW480 (N) cells. **(O-P)** Cell viability analysis after treatment with Terbinafine for 96 hours in DLD1 (O) and SW480 (P) cells with or without DOX-induced, ectopic *AP4* expression. **(Q-R)** Relative cholesterol concentration in DLD1 (Q) and SW480 (R) cells with or without DOX induced, ectopic *AP4* expression for 72 hours. In panel D, F, H, J and M-R the mean ± SD (n = 3) is provided. *: p < 0.05, **: p < 0.01, ***: p < 0.001, ****: p < 0.0001. TSS: Transcription start site.

Analysis of Encode ChIP-Seq datasets showed that the c-MYC-inducible transcription factor AP4 also binds to the *SQLE* regulatory region (Figure 8A). Moreover, we identified a putative AP4 binding motif in the *SQLE* promoter region (Figure 8B). A qChIP assay confirmed enhanced AP4 occupancy at this binding site within the *SQLE* promoter in SW480 cells (Figure 8C). Activation of a conditional *AP4* allele by DOX induced *SQLE* mRNA and protein levels in DLD1 and SW480 cells (Figure 8D-G). Furthermore, *AP4*-deficient cells showed a decrease in *SQLE* mRNA and protein expression (Figure 8H-K). Taken together, these results demonstrate that *SQLE* is directly induced by AP4.

Notably, analysis of DepMap data from CRC cell lines showed a positive association between dependencies on AP4 and SQLE (r=0.243) (Figure 8L). Therefore, we determined whether the *AP4* status influences the sensitivity of DLD1 and SW480 cells to Terbinafine. Indeed, *AP4*-proficient CRC cells were more sensitive to the suppression of SQLE when compared to *AP4*-deficient cells (Figure 8M and N), which were described before 20. Next, we analyzed the viability of DLD1 and SW480 cells with inducible *AP4* expression after Terbinafine treatment. Ectopic *AP4* further reduced cell viability when combined with Terbinafine treatment, suggesting that *AP4* sensitizes CRC cells to inhibition of cholesterol synthesis (Figure 8O and P). Finally, we determined that ectopic AP4 expression for 72 hours increased the levels of cholesterol (Figure 8Q and R). In summary, activation of AP4 induces SQLE expression and increases cholesterol levels, which ultimately sensitizes CRC cells to Terbinafine.

### Role of AP4 in the induction of SQLE expression by c-MYC

**Figure 9:**
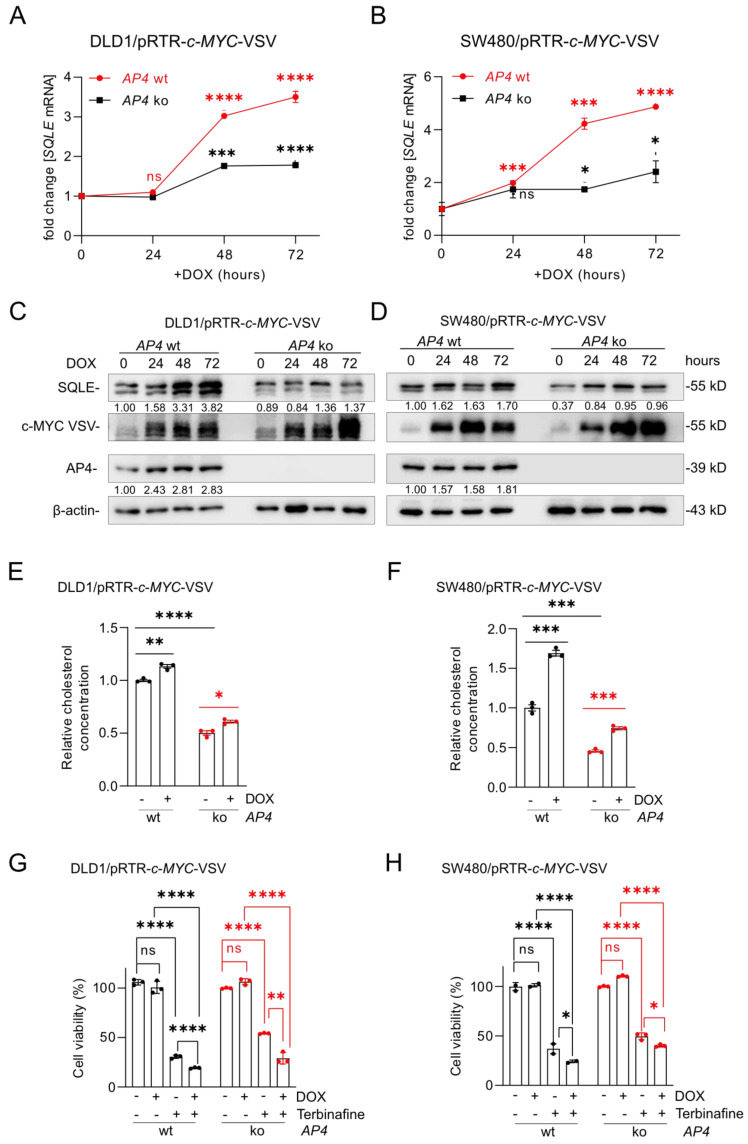
** Role of AP4 in the induction of *SQLE* by c-MYC. (A-B)** qPCR analysis of *SQLE* expression after c-*MYC* induction by DOX for the indicated periods in *AP4*-proficient and *AP4*-deficient DLD1 (A) and SW480 (B) cells. **(C-D)** Western blot analysis of SQLE protein expression after c-*MYC* induction by DOX for indicated periods in *AP4*-proficient and *AP4*-deficient DLD1 (C) and SW480 (D) cells. **(E-F)** Analysis of relative cholesterol concentration in *AP4*-proficient and *AP4*-deficient DLD1 (E) and SW480 (F) cells after c-*MYC* induction by DOX for 72 hours. **(G-H)** Cell viability analysis in *AP4*-proficient and *AP4*-deficient DLD1 (G) and SW480 (H) cells after c-*MYC* induction by DOX for 72 hours. In panel A, B and E-H the mean ± SD (n = 3) is provided. *: p < 0.05, **: p < 0.01, ***: p < 0.001, ****: p < 0.0001.

To analyze whether the induction of *SQLE* by c-MYC is mediated by AP4, we ectopically expressed c-MYC in *AP4*-proficient and *AP4*-deficient DLD1 and SW480 cells. *AP4*-deficiency largely prevented the induction of *SQLE* mRNA and protein expression after ectopic c-MYC. (Figure 9A-D). Consistent with these results, ectopic c-MYC increased cholesterol levels, which was attenuated in *AP4*-deficient CRC cells (Figure 9E and F). In addition, *AP4*-deficient cells showed significantly decreased levels of cholesterol levels. Ectopic c-MYC sensitized CRC cells to Terbinafine. However, this effect was less pronounced in *AP4*-deficient cells (Figure 9G and H). These results demonstrate that AP4 mediates the induction of *SQLE* by c-MYC. The resulting increase in cholesterol may explain the increased sensitivity of CRC cells with activated c-*MYC* to treatment with Terbinafine.

## Discussion

Here, we showed that c-MYC induces the expression of *SQLE* and thereby promotes cholesterol synthesis in CRC cells. Our results show that c-MYC induces *SQLE* expression largely indirectly via inducing its target gene *AP4* in CRC cell lines. AP4 is a member of the basic helix-loop-helix leucine zipper (bHLH-LZ) transcription factor family 33. We have previously shown that AP4 promotes stemness, EMT, migration, and invasion of CRC cells 34. c-MYC directly up-regulates AP4, which represses *p21*, to promote a proliferative, progenitor-like state 35, 36. Here we showed, that AP4 activation is sufficient to induce *SQLE* levels and promotes cholesterol synthesis. Taken together, these results imply that c-MYC and AP4 promote cholesterol synthesis and cancer cell proliferation through the induction of *SQLE*. The elevated cholesterol levels in *p53*-deficient CRC cells with high c-MYC expression presumably increase their dependency on cholesterol, which could explain the increased sensitivity of these cells to SQLE inhibition.

In this study, we identified SQLE as a vulnerability for CRCs with *p53* inactivation and elevated c-*MYC* expression. SQLE is key rate-limiting enzyme in the first oxygenation step in the cholesterol synthesis pathway 37. Cholesterol metabolism is required for cancer cell proliferation as cholesterol represents an essential membrane constituent that participates in various biological processes 38. As a component of cell membranes, cholesterol activates the oncogenic Hedgehog pathway by directly binding the Smoothened receptors 39, 40, which are closely related to cell differentiation and proliferation 41. Cholesterol is also an important component of lipid rafts, special small lipid domains within the cell membrane which serve as platforms for cellular signal transduction and oncogenic signaling pathways 42. Hence, cholesterol metabolism plays a crucial role in cancer cell proliferation. In addition, Costa et al. showed that cholesterol depletion suppresses the migration and invasion of melanoma cells 43. Elevated expression of *SQLE* has been associated with poor prognosis in CRC 44, 45, breast 46, 47, lung 48, and prostate cancer 49. Our results show that high *SQLE* expression is associated with poor prognosis particularly in patients with CRCs that harbor mutant *p53*.

c-MYC has been shown to activate the mevalonate/cholesterol pathway in cooperation with the E-box-binding B-HLH-LZ transcription factor SREBP1 to promote tumorigenesis in renal, hepatocellular, and leukemic mouse models 50. Moreover, SREBP-2 induces *c-MYC* expression by directly interacting with an SREBP-2-binding element in the 5'-flanking *c-MYC* promoter region 51. c-MYC expression is associated with increased in- and reduced e-flux of cholesterol in lung cancer 52. Therefore, multiple connections between c-MYC and cholesterol may play a role in cancer.

**Figure 10:**
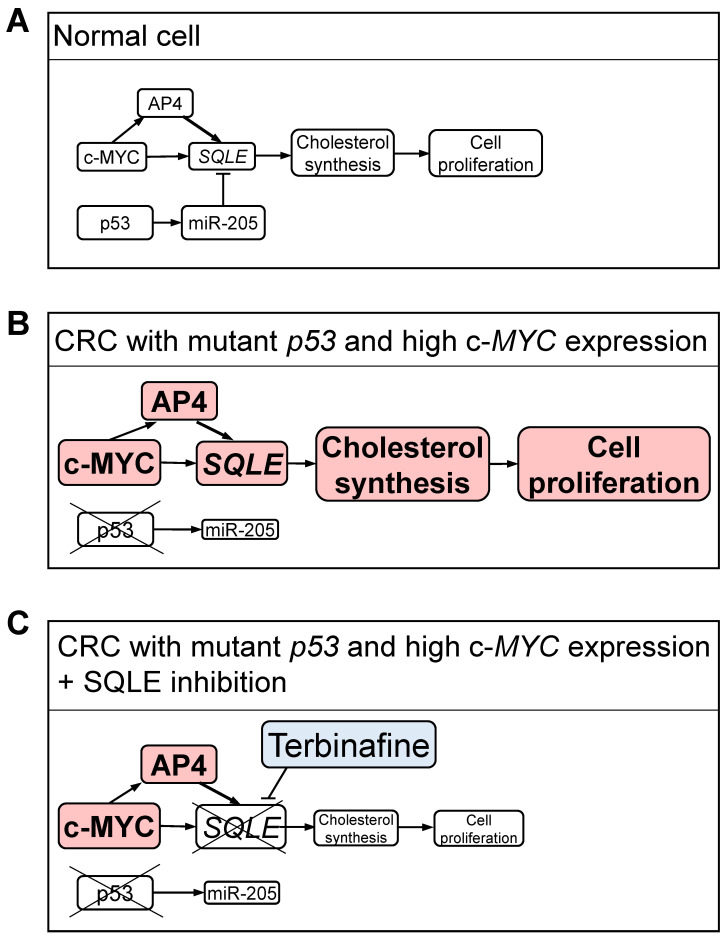
** Regulation and therapeutic inhibition of SQLE in CRC cells. (A)** In normal cells, wt p53 and low expression of c-MYC maintain low levels of *SQLE* and cholesterol resulting in controlled cell proliferation. **(B)** In CRC cells *p53* mutation, the resulting decrease in miR-205, and high c-*MYC* expression lead to an increase in *SQLE* expression and elevated cholesterol levels, which results in increased cell proliferation. **(C)** Inhibition of SQLE results in low cholesterol levels and decreased proliferation in CRC cells. A-C: Different levels of activity and/or expression are indicated by varying sizes of letters and rectangles.

Here, we showed that p53 represses *SQLE* expression via inducing miR-205. Consistently, the loss of *p53* resulted in elevated levels of cholesterol in three different CRC cell lines. Sun et al. proposed that p53 directly represses the expression of *SQLE* via binding to the first intron of the *SQLE* gene 53. However, our results imply that the repression of *SQLE* by p53 is mainly mediated by miR-205. While this study was ongoing others reported that *SQLE* represents a target of miR-205 54, which is a p53-inducible microRNA 31. However, these authors did not determine whether p53 represses *SQLE* via inducing miR-205, as shown here by us. Our findings are consistent with the notion that p53-mediated transcriptional repression is mediated by indirect mechanisms, such as induction of microRNA encoding genes or activation of the repressive DREAM complex via the induction of p21 55. Noteworthy, a recent meta-analysis of p53-regulated genes indicated that *SQLE* is not a target of the DREAM complex 29. As we show here, the induction of *miR-205* by p53 mediates the repression of *SQLE* by p53 to a large extent. Down-regulation of miR-205 has been observed in the majority of metastatic CRCs and is of predictive value for CRC metastasis 56. Down-regulation of miR-205 has also been observed in prostate, breast and liver cancers and is associated to tumorigenesis, cell proliferation, migration, invasion, and motility/metastasis 57-59. The regulatory connections between p53, c-MYC and *SQLE* provide a plausible explanation as to why SQLE represents a vulnerability in cancers with mutant *p53* and high c-MYC activity (Figure 10). Accordingly, inhibition of SQLE by Terbinafine particularly reduced viability in CRC cells with *p53* inactivation, high c-MYC expression and *AP4*-proficiency. Interestingly, Terbinafine was shown to induce cell cycle arrest and apoptosis in tumor cells, and inhibits angiogenesis by suppressing endothelial cell proliferation 60, 61. Since Terbinafine has been approved for antifungal therapy and has an excellent safety profile it may be quickly adapted for cancer therapeutic purposes 60, 62. Here, inhibition of SQLE effectively decreased viability in cultured CRC cells. However, CRC cells were cultured in medium supplemented with fetal bovine serum (FBS), which has a very low cholesterol concentration (0.09 mM at 10% FBS) 63. In humans, the concentration of cholesterol, which is produced in the liver, is much higher in plasma (5 mM) 63. Therefore, inhibition of SQLE might have a weaker effect *in vivo*. Yet, systemic inhibition of SQLE would also suppress cholesterol production in the liver. Noteworthy, treatment of mice with the SQLE inhibitor Terbinafine significantly inhibited the growth of hepatocellular cancer and CRC cells in xenograft models 45, 64, suggesting that the inhibition of SQLE may be a promising strategy for cancer treatment. However, when cholesterol synthesis is pharmaceutically inhibited, dietary cholesterol might represent the majority of blood cholesterol. Therefore, the effect of dietary cholesterol on therapies based on inhibition of SQLE should be determined in the future. In conclusion, we show that SQLE is up-regulated in CRCs with *p53* inactivation and elevated c-MYC/AP4 expression as it is directly and/or indirectly regulated by these factors. SQLE may therefore serve as a potential therapeutic target for treatment of CRCs with *p53* inactivation and elevated c-MYC/AP4 expression in the future.

## Supplementary Material

Supplementary figures and tables.
