# Supplementary material for: Squalene epoxidase/SQLE is a candidate target for treatment of colorectal cancers with p53 mutation and elevated c-MYC expression
Source: Int J Biol Sci. 2023 Aug 6;19(13):4103–22. doi: 10.7150/ijbs.85724 (PMC10496509; doi:10.7150/ijbs.85724)
Supplement: Supplementary file 1 — Supplementary figures and tables. [file ijbsv19p4103s1.pdf]

SUPPLEMENTAL MATERIAL

Squalene epoxidase/SQLE is a candidate target for treatment of colorectal cancers with *p53* mutation and elevated c-*MYC* expression

Yuyun Du[1,+], Matjaz Rokavec[1,+], and Heiko Hermeking[1,2,3]

[1] Experimental and Molecular Pathology, Institute of Pathology, Ludwig-Maximilians-Universität München, Germany

[2] German Cancer Consortium (DKTK), Partner site Munich, Germany

[3] German Cancer Research Center (DKFZ), Heidelberg, Germany

+: these authors contributed equally to this work

*: correspondence to: Dr. Heiko Hermeking, Experimental and Molecular Pathology, Institute of Pathology, Ludwig-Maximilians-Universität München, Thalkirchner Strasse 36, D-80337 Munich, Germany; e-mail: heiko.hermeking@med.uni-muenchen.de

Inventory of Supplemental Information

Supplemental Figure 1: Cell viability of HCT116, RKO and SW48 *p53*+/+ and *p53*-/- cells in the absence of Terbinafine

Supplemental Figure 2: Cell number of human normal intestinal fibroblasts after suppression of *SQLE*

Supplemental Figure 3: *SQL*E is repressed by *p53* and represents a potential target of the *p53*-induced microRNA-205

Supplementary Figure 4: *SQLE* expression is induced by c-MYC

Supplemental Table 1: Sequence information for miR-205-5p mimics and antagomirs

Supplemental Table 2: List of antibodies

Supplemental Table 3: Oligonucleotides used for qPCR

Supplemental Table 4: Oligonucleotides used for cloning and mutagenesis of *SQLE* 3'UTR

Supplemental Table 5: Oligonucleotides used for qChIP

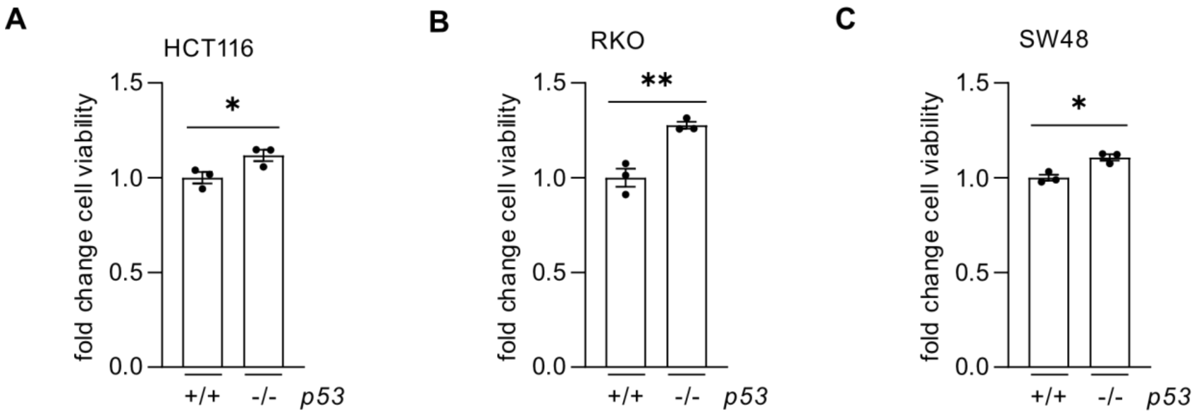

Supplemental Figure 1: Cell viability of HCT116 (A), RKO (B) and SW48 (C) *p53*+/+ and *p53*-/- cells in the absence of Terbinafine.

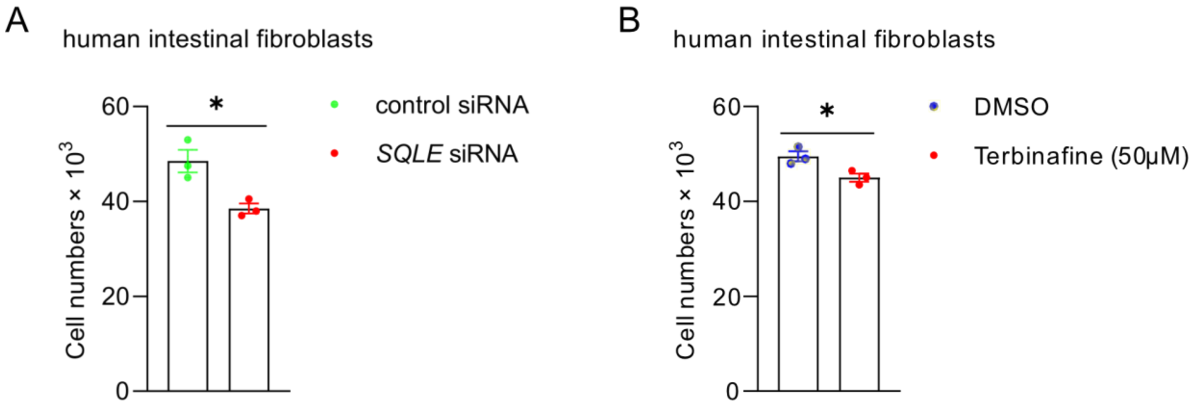

Supplementary Figure 2: Cell proliferation/number of human intestinal fibroblasts after knockdown of *SQLE* or treatment with the SQLE-inhibitor Terbinafine for 96 hours.

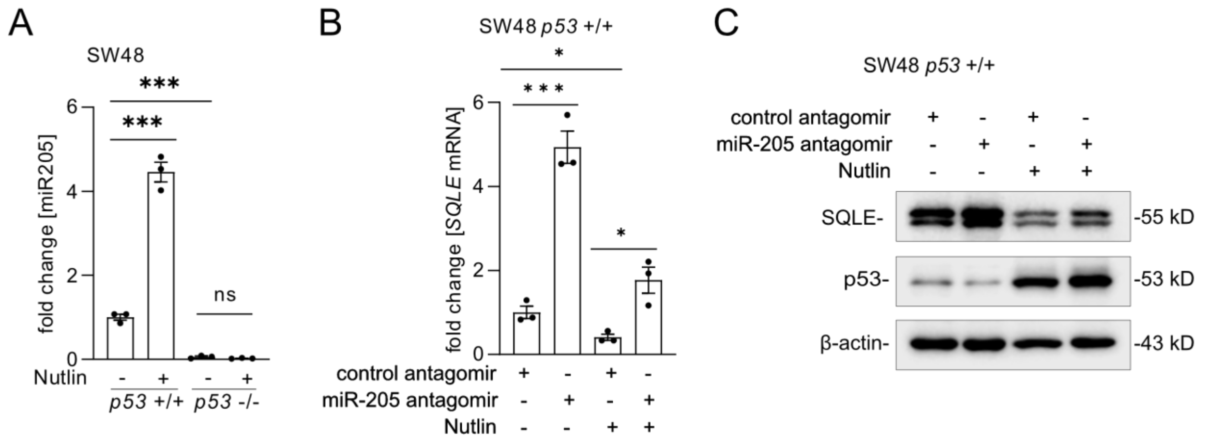

Supplemental Figure 3: *SQLE* is repressed by *p53* and represents a potential target of the *p53*-induced microRNA-205. **(A)** The expression of *SQLE* mRNA after the activation of *p53* by Nutlin-3a in SW48 cells. **(B)** qPCR analysis after transfection of miR-205 antagomir for 60 hours and Nutlin-3a treatment for 48 hours in SW48 cells. **(C)** Western blot analysis after transfection of miR-205 antagomir for 60h and Nutlin-3a treatment for 48h in SW48 cells.

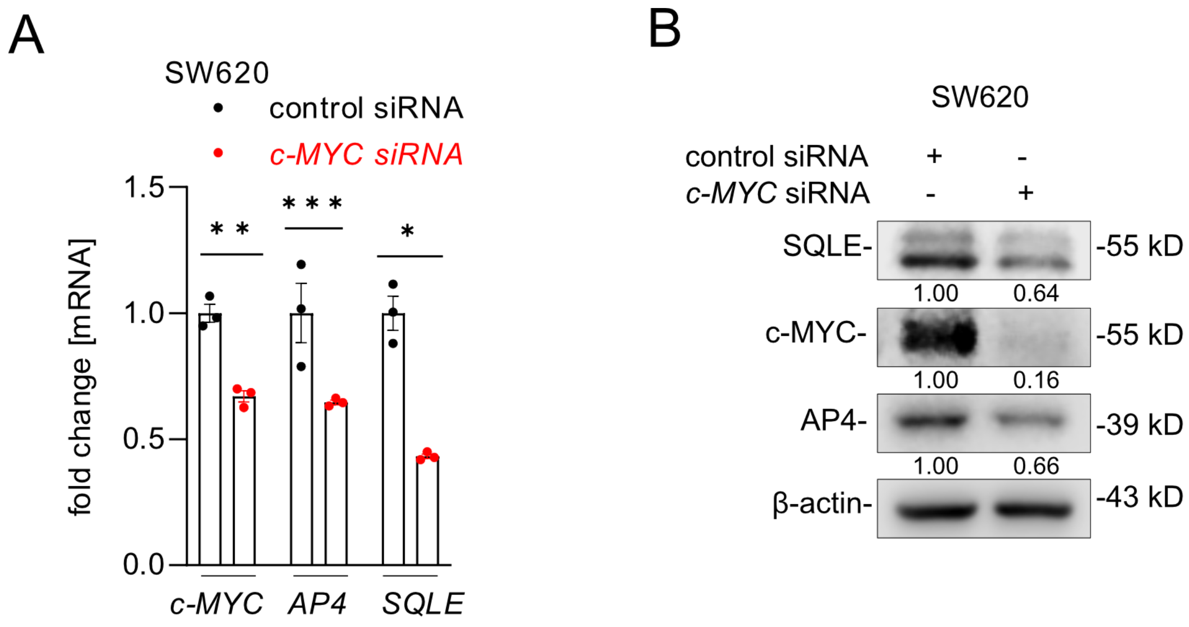

Supplemental Figure 4: *SQLE* expression is induced by c-MYC. **(A)** qPCR analysis after transfection of transfected with the indicated oligonucleotides in SW620 cells. **(B)** Western blot analysis after transfection of transfected with the indicated oligonucleotides in SW620 cells.

Supplemental Table 1: Sequence information for miR-205-5p mimics and antagomirs

Oligo	Sequence information (5‘-3‘)
hsa-miR-205-5p mimic	UCCUUCAUUCCACCGGAGUCUG
Control mimic	UCACCGGGUGUAAAUCAGCUUG
hsa-miR-205-5p antagomir	AGACTCCGGTGGAATGAAGG
Antagomir control	TAACACGTCTATACGCCCA

Supplemental Table 2: List of antibodies

Epitope	Species	Catalog No.	Compamy	Use	Dilution	Sourse
Primary antibodies						
SQLE	Human	#sc-271651	SANTA CRUZ	WB	1:1000	mouse
c-Myc	Human	#sc-764	SANTA CRUZ	WB	1:1000	rabbit
c-Myc	Human	#10828-1-AP	Proteintech	qCHIP	1:50	rabbit
AP-4 (A-8)	Human	#sc-377042	SANTA CRUZ	WB	1:1000	mouse
AP-4 (C-18)	Human	#sc-18595	SANTA CRUZ	qCHIP	1:50	goat
p53 (DO-1)	Human	#sc-126	SANTA CRUZ	WB	1:1000	mouse
β-actin	Human	# A2066	Sigma-Aldrich	WB	1:1000	rabbit
VSV	Human	# V4888	Sigma-Aldrich	WB	1:7500	rabbit
Second antibodies						
Anti-mouse HRP	N.A.	# W4021	Promega	WB	1:10000	goat
Anti-rabbit HRP	N.A.	# A0545	Sigma-Aldrich	WB	1:10000	goat

Supplemental Table 3: Oligonucleotides used for qPCR

gene	forward (5’-3’)	reverse (5’-3’)
GAPDH	TGTTGCCATCAATGACCCCTT	CTCCACGACGTACTCAGCG
β-actin	TGACATTAAGGAGAAGCTGTGCTAC	GAGTTGAAGGTAGTTTCGTGGATG
SQLE	GGCATTGCCACTTTCACCTAT	GGCCTGAGAGAATATCCGAGAAG
c-MYC	GGTGCTCCATGAGGAGACAC	GCCTGCCTCTTTTCCACAG
AP4	GCAGGCAATCCAGCACAT	GGAGGCGGTGTCAGAGGT

Supplemental Table 4: Oligonucleotides used for cloning and mutagenesis of *SQLE* 3’UTR

Oligo	Forward (5‘-3‘)	Reverse (5‘-3‘)
SQLE 3‘UTR	GAAGTTTTTTGTATATAAATATGTAAATACATGC	CTGTAGTTCTCCTCAGCATTAGCACT
SQLE 3’UTR mutant 1	TTAAACCGTCCTTGGTTAAATAAGTTAGACATTTAAAAGAAATGA	TAACCAAGGACGGTTTAAATTGCAAATTAAAGCATGTATTT
SQLE 3’UTR mutant 2	GAGACTTTtGGAAGAGGATATATATAGCATAGTACCATACC	CCTCTTCCaAAAGTCTCTTAGGACTTGGTAAGTTCC

Supplemental Table 5: Oligonucleotides used for qChIP

Gene	Forward (5’-3’)	Reverse (5’-3’)
SQLE (c-MYC bind site)	GCTATGCCGCGTTTGGCCAATC	CCTGAGCCCCGCCCCCGGTCCC
SQLE (AP4 bind site)	ATGGGCGGACTCGCGCCT	CTCACCAGCATCCCTCGCG
AP4 intron 1	GAGGTGGGCGTTCTACGG	GGTTGGGCAGGAGTGTCTAC
SNAIL	TTAAAATGCTGATGCCAGAGG	GCACATTAGATCCCCACAGG
16q22	CTACTCACTTATCCATCCAGGCTAC	ATTTCACACACTCAGACATCACAG
